# Cannabidiol Treatment Improves Glucose Metabolism and Memory in Streptozotocin-Induced Alzheimer’s Disease Rat Model: A Proof-of-Concept Study

**DOI:** 10.3390/ijms23031076

**Published:** 2022-01-19

**Authors:** Daniele de Paula Faria, Larissa Estessi de Souza, Fabio Luis de Souza Duran, Carlos Alberto Buchpiguel, Luiz Roberto Britto, José Alexandre de Souza Crippa, Geraldo Busatto Filho, Caroline Cristiano Real

**Affiliations:** 1 Laboratory of Nuclear Medicine (LIM 43), Department of Radiology, Faculdade de Medicina, University of Sao Paulo, Sao Paulo 05403-911, SP, Brazil; larissa.estessi@gmail.com (L.E.d.S.); buch@usp.br (C.A.B.); 2 Laboratory of Psychiatric Neuroimaging (LIM 21), Department of Psychiatry, Faculdade de Medicina, University of Sao Paulo, Sao Paulo 05403-911, SP, Brazil; fabio.duran@hc.fm.usp.br (F.L.d.S.D.); geraldo.busatto@hc.fm.usp.br (G.B.F.); 3 Institute of Biomedical Science, University of Sao Paulo, Sao Paulo 05508-000, SP, Brazil; britto@icb.usp.br; 4 Department of Neuroscience and Behavioral Sciences, Ribeirão Preto Medical School, University of Sao Paulo, Ribeirão Preto 14051-160, SP, Brazil; jcrippa@fmrp.usp.br

**Keywords:** cannabidiol, Alzheimer’s disease, streptozotocin, [^18^F]FDG PET imaging, glucose metabolism

## Abstract

An early and persistent sign of Alzheimer’s disease (AD) is glucose hypometabolism, which can be evaluated by positron emission tomography (PET) with ^18^F-2-fluoro-2-deoxy-D-glucose ([^18^F]FDG). Cannabidiol has demonstrated neuroprotective and anti-inflammatory properties but has not been evaluated by PET imaging in an AD model. Intracerebroventricular (icv) injection of streptozotocin (STZ) is a validated model for hypometabolism observed in AD. This proof-of-concept study evaluated the effect of cannabidiol treatment in the brain glucose metabolism of an icv-STZ AD model by PET imaging. Wistar male rats received 3 mg/kg of STZ and [^18^F]FDG PET images were acquired before and 7 days after STZ injection. Animals were treated with intraperitoneal cannabidiol (20 mg/kg—STZ–cannabidiol) or saline (STZ–saline) for one week. Novel object recognition was performed to evaluate short-term and long-term memory. [^18^F]FDG uptake in the whole brain was significantly lower in the STZ–saline group. Voxel-based analysis revealed a hypometabolism cluster close to the lateral ventricle, which was smaller in STZ–cannabidiol animals. The brain regions with more evident hypometabolism were the striatum, motor cortex, hippocampus, and thalamus, which was not observed in STZ–cannabidiol animals. In addition, STZ–cannabidiol animals revealed no changes in memory index. Thus, this study suggests that cannabidiol could be an early treatment for the neurodegenerative process observed in AD.

## 1. Introduction

Alzheimer’s disease (AD) has a multifactorial nature and is the most common neurodegenerative disease, being the main cause of dementia in the world [1]. Animal models have been used to understand the disease mechanisms. Streptozotocin (STZ), a glucosamine–nitrosourea compound [2] derived from soil bacteria and originally developed as an anticancer agent, has been used for diabetes models in animals since 1963. In the mid-1990s, the suggestion was raised that an intracerebroventricular (icv) injection of STZ could be an option to develop an animal model for AD. icv-STZ injection can decrease cerebral glucose uptake and produce multiple other effects that resemble the molecular, pathological, and behavioral features of the sporadic form of AD, including neuroinflammation. The glucose hypometabolism and desensitizing brain insulin receptors, observed in an icv-STZ model, are an early and persistent sign of sporadic AD and can be evaluated by positron emission tomography (PET) with ^18^F-2-fluoro-2-deoxy-D-glucose ([^18^F]FDG) [3]. Sporadic AD has been recognized as an insulin-resistant brain state (IRBS), and cerebral glucose metabolism has been the focus of preclinical testing of therapeutic interventions [2].

Cannabidiol (CBD) is a cannabinoid of the plant *Cannabis sativa* and has been suggested as a potential drug intervention for neurodegenerative diseases. The cannabinoid system can modulate cellular and molecular mechanisms, including excitotoxicity, oxidative stress, apoptosis, and inflammation, promoting neuroprotection in animal models. Mice inoculated with human Abeta (1–42) peptide into the right dorsal hippocampus and treated daily with CBD for 7 days showed a significant reduction in Aβ protein expression. In addition, an in vivo, anti-inflammatory effect was demonstrated, with lower iNOS and IL-1 beta protein expression [4]. On the other hand, an AD transgenic model (AβPPSwe/PS1ΔE9 (AβPP × PS1) mice) treated with CBD daily over the period of 8 months did not observe effects on soluble and insoluble Aβ40 or Aβ42 in the cortex and in the hippocampus of the animals but prevented the development of social recognition memory deficits [5]. Treatment for 7 days with cannabinoid type 1 (CB1)-selective receptor agonist arachidonyl-2′-chloroethylamide (ACEA) showed a reversed cognitive impairment and increased anti-apoptotic protein levels in a rat icv-STZ model, and rescue cells from STZ-triggered death and modulated NO release in an in vitro, neuronal model (Neuro-2a neuroblastoma cells) [6]. Despite the evidence of the beneficial effects of the cannabinoid system on neurodegenerative diseases, the use of CBD in icv-STZ has not yet been evaluated, and many doubts remain in the field. Thus, the aim of this proof-of-concept study was to evaluate by [^18^F]FDG PET the effect of cannabidiol treatment for 7 days on brain glucose metabolism and cognitive function in an icv-STZ rat model.

## 2. Results

### 2.1. Blood Glucose Level and Body Weight

The icv-STZ injection did not alter the blood glucose concentration (F(2,21) = 1.278; *p* = 0.2995; baseline—148 ± 23, STZ—126 ± 20 and STZ + CBD—134 ± 44).

**Figure 1:**
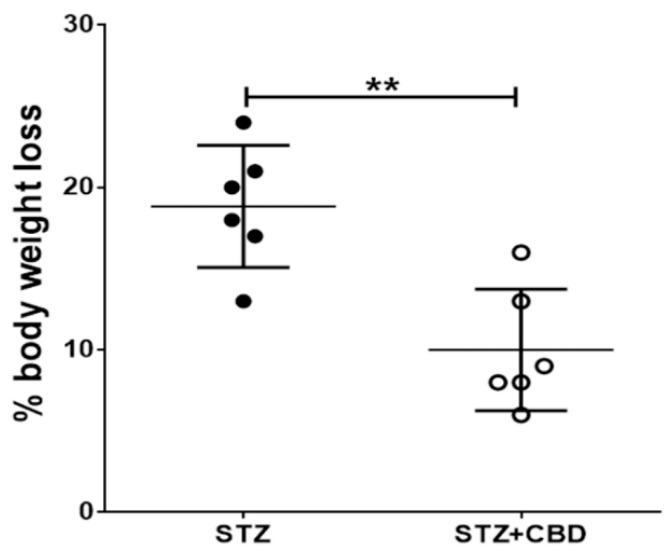
Comparison of percentage of weight loss (difference from the beginning to the end of the experiment) between non-treated (STZ) and treated animals (STZ + CBD). ** *p* < 0.01. Acronyms: STZ: streptozotocin, CBD: cannabidiol.

Animals from the STZ group revealed higher body weight loss (18.83% ± 1.54) from the surgery day to the final image when compared to STZ + CBD animals (10.0% ± 1.53, *p* = 0.0022), Figure 1.

### 2.2. Novel Object Recognition

**Figure 2:**
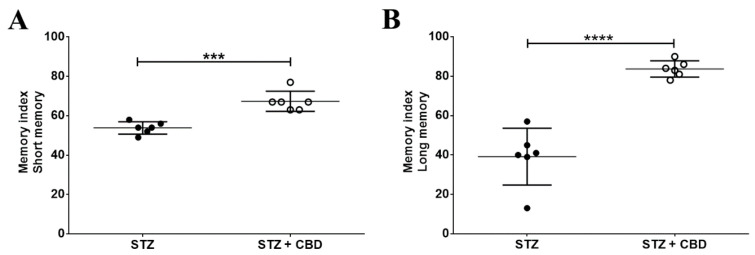
Memory index from the novel object recognition test: (**A**) Short memory index and (**B**) Long memory index. Acronyms: STZ: streptozotocin, CBD: cannabidiol. *** *p* < 0.001; **** *p* < 0.0001.

The novel object recognition test revealed a worse memory index for STZ animals, both for short-term (53.83 ± 3.12, −20%) and long-term memory (39.17 ± 14.43, −53%), when compared to STZ + CBD animals (SM—67.33 ± 5.13, *p* = 0.0003; LM—83.67 ± 4.13, *p* < 0.0001), Figure 2.

### 2.3. [^18^F]FDG PET Imaging

**Figure 3:**
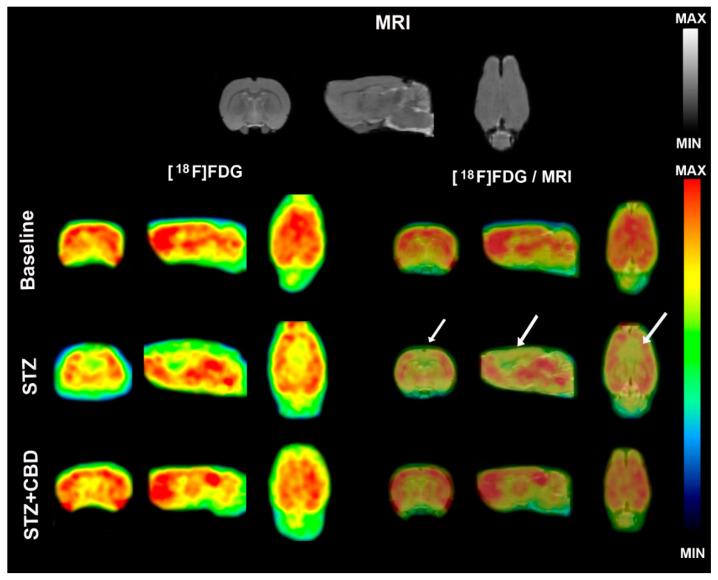
Illustrative [^18^F]FDG PET images. MRI template used in the analysis is shown in the top of the figure. [^18^F]FDG PET images are shown for baseline (first line), streptozotocin animals without treatment (STZ—second line), and STZ animals treated with cannabidiol (STZ + CBD—third line). Left side of the figure shows only the [^18^F]FDG PET images, and the right side shows the PET images fused to the MRI template. White arrows show the areas with hypometabolism. Acronyms: STZ: streptozotocin, CBD: cannabidiol, [^18^F]FDG: ^18^F-2-fluoro-2-deoxy-D-glucose, MRI: magnetic resonance imaging.

**Figure 4:**
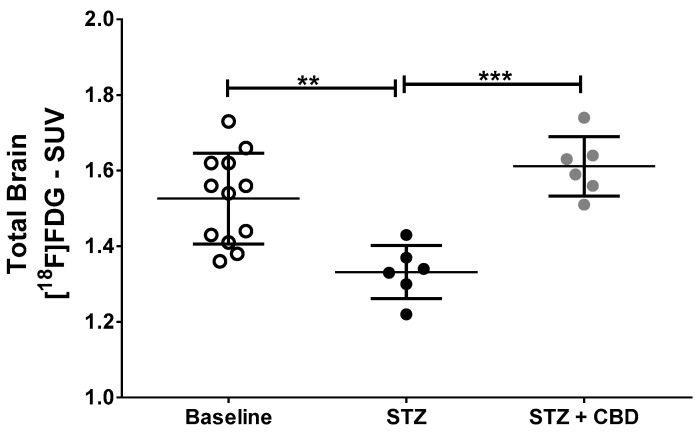
[^18^F]FDG uptake (SUV—standardized uptake value) in the whole brain. Comparison of baseline (*n* = 12), non-treated animals (STZ, *n* = 6), and cannabidiol-treated animals (STZ + CBD, *n* = 6). ** *p* < 0.01; *** *p* < 0.001. Acronyms: STZ: streptozotocin, CBD: cannabidiol, SUV: standardized uptake value, [^18^F]FDG: ^18^F-2-fluoro-2-deoxy-D-glucose.

[^18^F]FDG PET image data are described in Table 1 and showed, in general, lower uptake in the analyzed brain areas of STZ animals when compared to baseline and STZ + CBD uptake. Figure 3 and Figure 4 show illustrative [^18^F]FDG PET images and the standardized uptake value (SUV) in the whole brain, respectively.

### 2.4. Small Animal Molecular Imaging Toolbox

**Figure 5:**
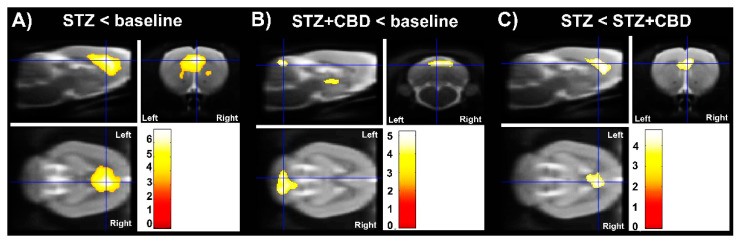
Voxel-based analysis. Lower [^18^F]FDG uptake was observed after intracerebroventricular streptozotocin injection (STZ), less significant in animals treated with cannabidiol (CBD). Significance is shown with a T statistic color scale, which corresponds to the level of significance at the voxel level. The data were derived from 12 rats for baseline and 6 rats for experimental groups, being: (1) intracerebroventricular streptozotocin injection (STZ) and (2) cannabidiol treatment for 7 days after STZ (STZ + CBD). (**A**) Hypometabolism in STZ group when compared to baseline data. (**B**) Hypometabolism in STZ + CBD group when compared to baseline data. (**C**) Hypometabolism in STZ group when compared to STZ + CBD group. Acronyms: STZ: streptozotocin, CBD: cannabidiol.

The results of the voxel-based analysis are shown in Figure 5 and Table 2. Uptake of [^18^F]FDG was significantly lower in STZ rats than in baseline scans and STZ + CBD rats (cluster-level *p*  <  0.05, corrected for family-wise error).

## 3. Discussion

This was a proof-of concept study using [^18^F]FDG PET imaging as a tool to evaluate the effect of cannabidiol in the icv-STZ rat model of Alzheimer’s disease. Our results showed that [^18^F]FDG PET imaging was able to detect glucose hypometabolism in the brain of animals with induced icv-STZ and that cannabidiol had a protective effect over the STZ action. Cannabidiol was also able to avoid short- and long-term memory damage in the STZ animal model. As far as we know, this is the first study using [^18^F]FDG PET imaging to evaluate cannabidiol treatment in the icv-STZ-induced AD animal model.

The STZ animal model is considered an Alzheimer’s disease animal model due to STZ brain effects after intracerebroventricular injection, which is related to the hypothesis that there is an association between type 2 diabetes and AD [2,7]. There is a direct effect of icv-STZ injection on memory, where there is memory disruption just a few hours after the STZ injection [8]. Our findings also showed memory deficit after STZ injection; however, the short- and long-term memory was preserved by the cannabidiol treatment compared to animals without treatment.

The animals from the STZ group presented significant weight loss compared to the animals treated with cannabidiol. The weight loss was already expected in this animal model and was explained by Poddar et al. [9] as a consequence of a decrease in complex I-III activity. In our treated group the weight loss was approximately 10% compared to baseline, which could be a consequence of the surgery procedure itself.

Xiong and Lim [10], in a recent review, discussed the effects of cannabidiol on AD and presented some possible action mechanisms for this drug: among them, an increase in neurogenesis, decrease in Aβ burden, inflammation and oxidative damage, and improvement in cognitive function which was already shown in AD mice [11]. One of these mechanisms, or all of them, could be responsible for our findings. CBD has already shown anxiolytic, neuroprotective, antidepressant, anti-inflammatory, and immunomodulating effects [12] and also has the potential to be used in combination with other drugs already used in the treatment of AD [13].

[^18^F]FDG PET imaging is a tool to evaluate glucose metabolism and, in the case of neurodegenerative diseases, such as Alzheimer’s disease, this tool can detect hypometabolism in some brain regions that have a direct correlation with neurodegeneration. Since [^18^F]FDG is a glucose analogue and its uptake, especially brain uptake, can be changed by the blood glucose level [14], it is important to emphasize that the animals in this study presented no alterations in this parameter, which could be explained by the STZ administration route (intracerebroventricular instead of systemic) and is in agreement with the literature [15].

[^18^F]FDG PET imaging was able to show a decrease in brain metabolism after icv-STZ injection, as well the protective effect of cannabidiol, which suggests the important role of this imaging technique in the evaluation of treatments for AD. The hypometabolism observed by [^18^F]FDG PET in our AD model corroborates a previous study [3]. In addition, a recent study [16] used this imaging method in the STZ animal model and, although the authors studied a diabetes model (intraperitoneal STZ injection), and the focus was on sex differences in brain metabolism and not treatment, they also found lower brain uptake in the diabetic rats compared to control animals.

Although STZ changes glucose metabolism systemically, its action is through GLUT2 transporter, which is not present in the blood–brain barrier (BBB) and, therefore, for action in the brain, the STZ needs to be injected intracerebroventricularly [2]. [^18^F]FDG crosses the BBB by GLUT1 [7] transporters, meaning that there is no influence or competition of the tracer entrance and the STZ.

The voxel-based analysis confirmed the results of VOI analysis where the hypometabolism is more evident in the STZ group compared to baseline and STZ + CBD, starting close to the lateral ventricle where the STZ was injected and including part of cortex and corpus callosum. An unexpected result was the hypometabolism in the region of the third ventricle and cerebellum in the group treated with the cannabidiol compared to baseline. This finding may be related to the migration of STZ injection and/or some action of CBD, for example, cerebellar deactivation [17].

This study has some limitations; the main one being the short period (1 week) of treatment. Previous studies [8,18] show that amyloid load increases from 2 weeks of STZ injection; therefore, this parameter was not measured in our study. There was also the treatment scheme, which could be tested as a chronic treatment (longer), as well as only an acute treatment. Due to the short period of evaluation and the intent to focus on the in vivo technique, we did not perform an in vitro technique, such as immunohistochemistry.

There is still a limited number of studies evaluating the status of brain glucose metabolism in the icv-STZ animal model [19]; therefore, this study is important in providing more data on this topic and also in showing the potential of cannabidiol treatment to improve memory and glucose metabolism in an AD animal model.

## 4. Materials and Methods

### 4.1. Animals

All procedures were performed according to the protocol approved by the Institutional Animal Care and Ethics Committee on Animal Use (CEUA) of the University of São Paulo Medical School (FMUSP, Brazil) (protocol number: 984/2018, permission code, 21 February 2018). Outbred, male Charles River Wistar rats (three months old, *n* = 12) were purchased from the central animal facility of the Institute of Biomedical Sciences of the University of São Paulo and group-housed in thermo-regulated (21 ± 2 °C) and humidity-controlled rooms. Food and water were available ad libitum. The rats were acclimatized for at least seven days before starting the experimental protocol. Animals were randomly divided into 2 groups (*n* = 6 each): (1) streptozotocin animals (STZ), which were injected bilaterally into the lateral ventricles with STZ; and (2) streptozotocin–cannabidiol animals (STZ + CBD), which were injected bilaterally into the lateral ventricles with STZ and treated with intraperitoneal CBD injection immediately after surgery until the end of the experiment (total of 1 week). Sample size calculation was performed with G*Power 3.1.9.2 software (Universität Düsseldorf, Germany) based on published data for [^18^F]FDG in other studies.

### 4.2. Intracerebroventricular Injection of Streptozotocin

The animals were anesthetized with isoflurane mixed with oxygen (5% induction, 2% maintenance, 0.8 L/min) and placed in a stereotaxic apparatus (David Kopf instruments, Tujunga, CA, USA). After craniotomy, two aliquots of 3 µL of STZ (3 mg/kg, icv—freshly dissolved in citrate buffer—0.05 mol/L, pH 4.5—S0130, Sigma, supplier: Merck Life Science ApS, Søborg, Denmark) were injected bilaterally into the lateral ventricles by a needle attached to the Hamilton^®^ syringe (Neuros Syringes—65460-02, Reno, NV, USA). The solution was injected into the lateral ventricles at the following coordinates: AP—0.8 mm; ML ± 1.4 mm; and V—3.4 mm relative to bregma and ventral to the dura mater [20], as previously described [6]. After slow infusion (0.5 µL/min), the syringe needle remained in the infused region for 3 min to avoid reflux of the solution. The incision was sutured. To reduce discomfort, pain medication (ketoprofen—1 mg/kg, s.c., Sanofi-Aventis Farmacêutica, Suzano, São Paulo, Brazil) was given during surgery and at 24 h after surgery. If the rats still showed signs of discomfort after 48 h, an extra dose of analgesic was given.

### 4.3. Intraperitoneal Administration of Cannabidiol

Half of the animals (*n* = 6) received intraperitoneal treatment of cannabidiol 20 mg/kg (Biosynthesis Pharma Group Limited—BSPG, Sandwich, UK) diluted in vehicle solution (2% Tween 80 and 98% saline) immediately after the surgical procedure and on the following 6 days (total of 1 week of treatment).

**Figure 6:**
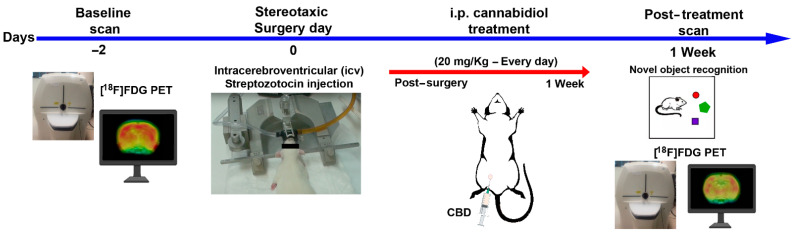
Scheme of study design.

The study design is represented by the scheme in Figure 6.

### 4.4. Novel Object Recognition (NOR) Behavioral Test

The NOR was carried out on day 6 after surgery. All objects used for the test had similar textures, colors, and sizes but distinctive shapes. The rats were placed in a rectangular arena (76 × 49 × 57 cm) containing two identical objects (A1 + A2) for the habituation and training phase, as previously described [21], but with some modifications. After 5 min, the rats were removed from the arena and placed in their home cage. After 1 h (short-term memory, SM) and 24 h (long-term memory, LM), the rats were placed in the arena again for 5 min, with one object replaced by a novel object (SM: B + A2, LM: C + A2). The behavioral tests were video recorded for further analysis. Exploration of an object is defined as the time the animal spends with its head oriented towards the object, within two centimeters of the object (sniffing). The discrimination index was calculated by:

[(time exploring the new object)/(total time exploring the two objects)] × 100%

### 4.5. PET Imaging

[^18^F]FDG was produced at the Nuclear Medicine Center, FMUSP. [^18^F]FDG PET images were acquired at baseline (before stereotactic surgery) and 1 week after surgery using a small-animal PET scanner (Triumph™—Gamma Medica-Ideas, Northridge, CA, USA).

Rats were anesthetized with isoflurane mixed with oxygen (5% induction, 2% maintenance, 0.8 L/min) and then injected into the penile vein with 37–55 MBq of [^18^F]FDG. Forty-five minutes after the tracer injection, the animals were positioned in the scanner with their heads in the center of the field of view. A static image was acquired for 30 min. The body temperature of the animals was maintained by heating pads, and the breathing rate was monitored, and eye lubricant was applied onto the eyes to prevent dehydration. After the scans, the animals were either allowed to recover in their home cages or euthanized (end of experiment).

Emission sinograms were iteratively reconstructed into a single frame of 30 min (OSEM 3D; 20 iterations and 4 subsets) after being normalized and corrected for scatter and radioactivity decay. PET image analysis was performed with PMOD software (PMOD™ Technologies Ltd., Zurich, Switzerland). [^18^F]FDG PET images were registered to MRI template, and volumes of interest (VOI), available on PMOD software, were applied for brain analysis.

The brain radioactivity concentration was calculated in each VOI and expressed as standardized uptake value (SUV): [tissue activity concentration (kBq/mL) × body weight (g)]/[injected dose (kBq)] for each region. A tissue density of 1 g/mL was assumed.

### 4.6. Voxel Based Analysis

Voxel-based analyses [22] were performed in Statistical Parametric Mapping, version 12 (SPM12; https://www.fil.ion.ucl.ac.uk/spm/ accessed on 21 December 2021), accessed/installed on 5 June 2019, using [^18^F]FDG data in combination with the Small Animal Molecular Imaging Toolbox (SAMIT; http://mic-umcg.github.io/samit/ accessed on 21 December 2021) package, accessed/installed on 5 June 2019.

All the images were spatially normalized using affine transformations to a tracer-specific rat brain PET template in Paxinos space [23] and smoothed with a 1.2 mm isotropic Gaussian kernel. To ensure that the analysis contained only voxels mapping the rat brain, a threshold of 0.5 of the mean radiotracer uptake in the rat brain was selected. The differences between the global uptake of images were adjusted using the proportional scaling option of SPM12.

For the interpretation of group differences, T map thresholds were set at ≤0.001, uncorrected for multiple comparisons, and the extent threshold ≥200 voxels for the cluster size (kE) (voxel size of 0.2 × 0.2 × 0.2 mm). Only clusters with *p* ≤ 0.05, corrected for family-wise error (FWE), were considered significant [24].

### 4.7. Statistical Analysis

Data are presented as mean and standard deviation (SD). Body weight and novel object recognition data were analyzed using the Student’s *t*-test. [^18^F]FDG data for each brain area and blood glucose level were analyzed by one-way ANOVA followed by Tukey’s multiple comparisons test. Differences were considered statistically significant when *p* < 0.05. All data were analyzed using GraphPad Prism 6 software (San Diego, CA, USA).

## 5. Conclusions

Our data suggest that cannabidiol treatment was able to protect the brain from STZ effects, which promoted a dysregulation in the glucose metabolism. The absence of alterations in the glucose metabolism could be important for maintaining cognitive ability, which was altered in the animals without treatment. Therefore, cannabidiol could be an important therapeutic option for Alzheimer’s disease, given that there is early and persistent glucose metabolism dysfunction in this disease, and [^18^F]FDG PET imaging may be a valuable tool to evaluate its effect.

## Figures and Tables

**Table 1:** [^18^F]FDG uptake (SUV) in different brain regions and statistical comparison between groups.

Brain Areas	SUV			F Value	*p* Value		
	Baseline	STZ	STZ + CBD		Baseline vs. STZ	Baseline vs. STZ + CBD	STZ vs. STZ + CBD
Total Brain	1.53 ± 0.12	1.33 ± 0.07	1.61 ± 0.08	F(2,21) = 12.38; *p* = 0.0003	*p* = 0.0026	*p* = 0.2291	*p* = 0.0003
Striatum	1.72 ± 0.10	1.48 ± 0.08	1.78 ± 0.11	F(2,21) = 15.13; *p* < 0.0001	*p* = 0.0003	*p* = 0.5102	*p* = 0.0002
Amygdala	1.19 ± 0.08	1.18 ± 0.13	1.31 ± 0.10	F (2,21) = 3.237; *p* = 0.0595	*p* = 0.9592	*p* = 0.0789	*p* = 0.0901
Frontal cortex	1.52 ± 0.20	1.30 ± 0.21	1.49 ± 0.16	F(2,21) = 2.82; *p* = 0.082	*p* = 0.073	*p* = 0.929	*p* = 0.2269
Hippocampus (anterodorsal)	1.62 ± 0.13	1.45 ± 0.13	1.73 ± 0.10	F(2,21) = 9.585; *p* = 0.001	*p* = 0.0141	*p* = 0.1719	*p* = 0.0009
Hippocampus (posterior)	1.41 ± 0.08	1.23 ± 0.10	1.42 ± 0.10	F(2,21) = 8.562; *p* = 0.0019	*p* = 0.0029	*p* = 0.9606	*p* = 0.0054
Hypothalamus	1.36 ± 0.10	1.31 ± 0.17	1.43 ± 0.18	F(2,21) = 1.13; *p* = 0.3427	*p* = 0.7261	*p* = 0.6092	*p* = 0.3125
Motor cortex	1.59 ± 0.16	1.36 ± 0.13	1.65 ± 0.10	F(2,21) = 7.203; *p* = 0.0042	*p* = 0.0103	*p* = 0.7284	*p* = 0.0061
Superior colliculus	1.71 ± 0.17	1.74 ± 0.22	1.74 ± 0.21	F (2,21) = 0.0832; *p* = 0.9205	*p* = 0.9394	*p* = 0.9424	*p* > 0.9999
Thalamus	1.70 ± 0.15	1.54 ± 0.15	1.77 ± 0.11	F (2, 21) = 4.34; *p* = 0.0264	*p* = 0.0817	*p* = 0.5798	*p* = 0.0253

Acronyms: STZ: streptozotocin, CBD: cannabidiol, SUV: standardized uptake value.

**Table 2:** Statistical parametric mapping outcome for the clusters.

	Cluster Level		Voxel Level		Coordinates			Brain Area
	P_FWE-corr_	k_E_	T	P_uncorr_	x	y	z	
Categorical design: [^18^F]FDG								
STZ < baseline	<0.001	6298	6.88	<0.001	0.8	1.6	−2.6	Regions close to lateral ventricle (anterior CPu, cortex, and cc)
STZ + CBD < baseline	0.017	1158	5.20	<0.001	−1.6	−1.8	−5.4	Regions close to dorsal 3rd ventricle (left hemisphere)
	0.024	1039	4.86	<0.001	1.8	−12.4	−2.4	6th cerebellar lobule
STZ < STZ + CDB	0.008	1484	4.73	<0.001	0.6	2.6	−4.0	Regions close to lateral ventricle (close to anterior CPu and cc)

P_FWE-corr_: the chance (*p*) of finding a cluster with this or a greater size, corrected for search volume. k_E_ = cluster size. T = outcome of statistical significance. P_uncorr_: the chance (*p*) of finding a voxel with this or a greater T value, uncorrected for search volume. x = medial–lateral direction—distance relative to bregma (negative values to the left side). y = anteroposterior distance relative to bregma (negative values to the posterior side). z = dorsoventral distance relative to bregma. Acronyms: STZ: streptozotocin, CBD: cannabidiol, [^18^F]FDG: ^18^F-2-fluoro-2-deoxy-D-glucose.

## Data Availability

The data that support the findings of this study are available from the corresponding authors upon reasonable request.
